# Laparoscopic resection of a paraganglioma behind the retrohepatic segment of the inferior vena cava: a case report and literature review

**DOI:** 10.3389/fendo.2023.1171045

**Published:** 2023-07-17

**Authors:** Wenda Wang, Jianhua Deng, Hanzhong Li, Zhigang Ji, Jin Wen

**Affiliations:** Department of Urology, Peking Union Medical College Hospital, Chinese Academy of Medical Sciences and Peking Union Medical College, Beijing, China

**Keywords:** paraganglioma, retrohepatic, inferior vena cava, laparoscopy, resection

## Abstract

**Background:**

Due to the location of paragangliomas (PGLs) behind the retrohepatic segment of inferior vena cava (IVC), it is difficult to expose and resect the tumor.

**Case presentation:**

A tumor measuring 50×45×62cm behind the retrohepatic portion of IVC was found in a 51-year-old female with hypertention and diabetes mellitus. Although the test for catecholamines revealed no signs of disease, the enhanced computed tomography (CT) scan, somatostatin receptor imaging and iodine-131-labeled metaiiodo-benzylguanidine (^131^I-MIBG) imaging revealed that the tumor was PGL. A three-dimensional printing was performed to visualize the tumor. The laparoscpic surgery for the PGL behind the retrohepatic segment of IVC was performed and the tumor was resected completely without causing any tissues injury. The pathologic diagnosis was PGL and the patient was able to recover well.

**Conclusions:**

This case demonstrates that laparoscopic surgery may be helpful in tumor accessibility, and could be used in the appropriate cases to remove PGLs that are located behind the retrohepatic segment of the IVC.

## Background

Paraganglioma/pheochromocytoma (PGL/PHEO) is a tumor originating in the chromaffin tissue of the neuroectoderm, which mainly secretes catecholamines (1). The classic features include headache, diaphoresis and tachycardia, occurring in only 24% of the cases (2). 90% of patients have hypertension, and the other clinical features include orthostatic hypotension, pallor, chest pain, dyspnea, weight loss, heat intolerance, nausea, vomiting, constipation and psychiatric disorders (3). 15-17% cases develops metastatic disease and extra-adrenal tumors have been associated with a higher risk for metastatic spread (4).

PGLs could be divided into parasympathetic PGLs (including chemoreceptor tumor, carotid body tumor, etc.) and sympathetic PGLs (including mediastinal, retroperitoneal and pelvic PGLs) depending on whether the tumor comes from the sympathetic or parasympathetic ganglia (5). Surgical resection is the first-line therapy to cure non-metastatic PGLs (6). When complete resection of the primary tumor is not possible or metastatic disease is present, tumor debulking may be used to relieve symptoms due to catecholamine excess (7).

However, the locations of some PGLs are very special, which brings great difficulties to surgical resection. There have been few reports of surgical treatment of PGLs behind inferior vena cava (IVC), but the locations are usually low (near renal veins) (8–12). Herein, we report a case of PGL behind the retrohepatic segment of IVC that was resected by laparoscopy.

## Case presentation

A 51-year-old middle-aged woman was hospitalized due to hypertension for 5 years. Blood pressure fluctuated between 150-160/100-110mmHg and could be controlled by medication. There were no accompanying symptoms of headache, dizziness, palpitation, and hyperhidrosis. The general condition was good.

The patient was diagnosed with diabetes mellitus nine years ago. fasting blood glucose (FBG) and postprandial blood glucose (PBG) fluctuated between 6-7mmol/L and 7-10mmol/L, respectively. She also had hyperlipidaemia for two years. The patient had a history of tonsillectomy. The patient had no special personal or family history.

**Figure 1:**
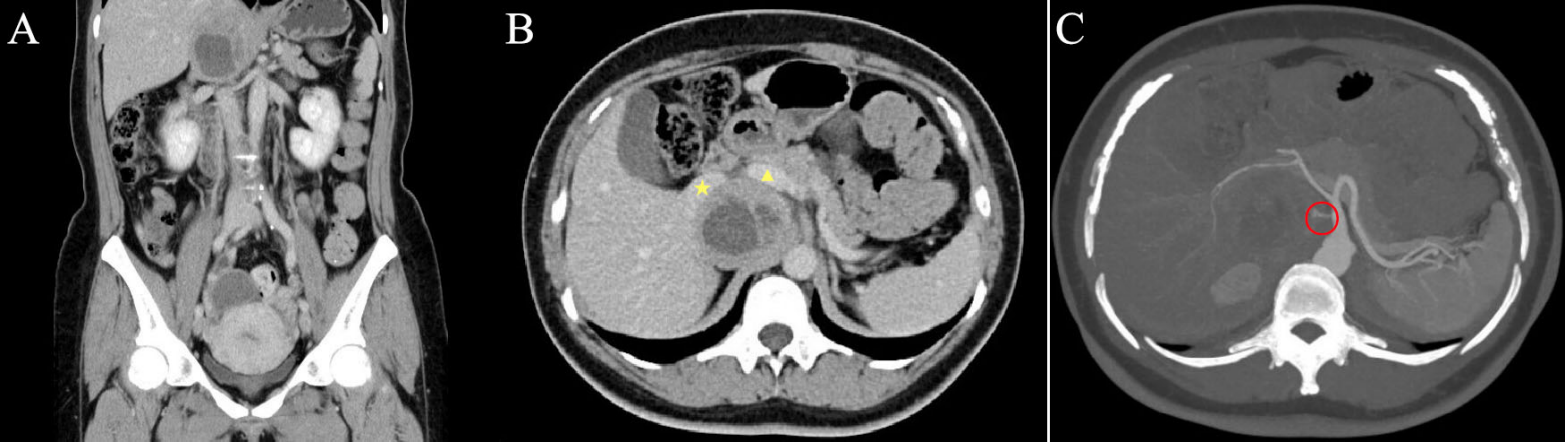
Contrast-enhanced computed tomography of abdomen and pelvis. **(A)** Three-dimensional reconstruction; **(B)** The solid cystic mass was between IVC and phrenic foot (★ IVC, ▴ portal vein); **(C)** There was an arterial vessel that originated from the aorta (red circle) supplying blood directly to the tumor.

**Figure 2:**
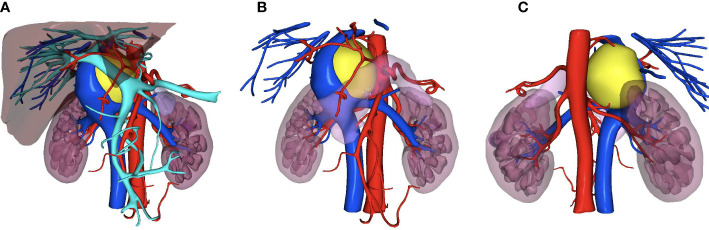
The three-dimensional printing for the tumor. **(A)** Front view; **(B)** Front view without liver; **(C)** Dorsal view. Yellow, tumor; Red, artery; Deep blue, IVC system; Light blue, portal vein system.

Physical examination showed no obvious positive signs. Laboratory examination showed normal blood levels of meta-norepinephrine (0.53 nmo1/L, normal range <0.9 nmol/L) and metanephrine (0.06 nmo1/L, normal range < 0.5nmol/L), and 24-hour urine catecholamine levels were as follows: Dopamine 310.6 μg/24 h (normal range <459.9 μg/24 h), norepinephrine 2.2 μg/24 h (normal range <11.0 μg/24 h), and epinephrine 31.9 μg/24 h (normal range <76.9 μg/24 h). The hemoglobin level declined (90g/L). Contrast-enhanced computed tomography (CT) of the abdomen and pelvis showed the following findings (**Figure 1**): There was a solid cystic mass in the right retroperitoneal space. The mass size was 50×45×62cm, and there was an obvious enhancement in the solid part. The mass was located between IVC and the phrenic foot. The IVC, right renal vein, and right and caudate lobes of the liver were significantly crushed. Somatostatin receptor imaging and iodine-131-labeled metaiiodo-benzylguanidine (^131^I-MIBG) imaging were also performed, and the solid-cystic mass showed increased radioactive uptake in the solid portion. To reproduce the tumor morphology more intuitively, our hospital carried out three dimensional (3D) printing based on imaging data (**Figure 2**).

Although the patient had no typical features or elevated catecholamines, the results of somatostatin receptor imaging and ^131^I-MIBG imaging supported the clinical diagnosis of PGL. The patient did not undergo germline mutational testing. The patient was treated for anemia, and the hemoglobin level was raised to 118g/L before surgery. The patient was also given medication (phenoxybenzamine) for 3 months prior to surgery. After consultation with liver surgery, vascular surgery, thoracic surgery, anesthesiology, and intensive care unit departments, our department and liver surgeons developed a special surgical plan for this case: Laparoscopic resection of the PGL.

The patient was placed in the supine position. The intraperitoneal route was adopted, and laparoscopic puncture holes were chosen as: 1) parumbilical, 2) 5cm above umbilicus, 3) 2cm inside the right anterior superior spine, 4) below the xiphoid, 5) subcostal boundary of the right midclavicular line. The right lateral peritoneum was opened, and the ascending colon was isolated to expose the tumor area. The tumor was completely covered by IVC (**Supplementary Figure 1**). Vessel loops were used for direction and traction of important large vessels, including IVC, left renal vein and portal vein, and the tumor was gradually exposed (**Supplementary Figures 2**, **3**). Then the tumor was carefully separated from them. The retrohepatic segment of IVC was isolated from the tumor surface by ligation of several short hepatic veins. The tumor’s regurgitant veins were severed (**Supplementary Figure 4**). For dense adhesion of the right adrenal gland with the tumor, the adrenal gland was also excised (**Supplementary Figure 5**). The tumor was then carefully separated from surrounding tissues. The arterial vessel from the aorta, which supplied blood directly to the tumor, was also cut off (**Supplementary Figure 6**). Finally, the tumor with a complete envelope was removed (**Supplementary Figures 7**, **8**). The operation lasted 340 min, and the blood loss was about 200 ml. The patient recovered well after the operation without any serious complications. Oral intake and ambulation were resumed on postoperative day 1, and the patient was discharged from the hospital on postoperative day 8.

**Figure 3:**
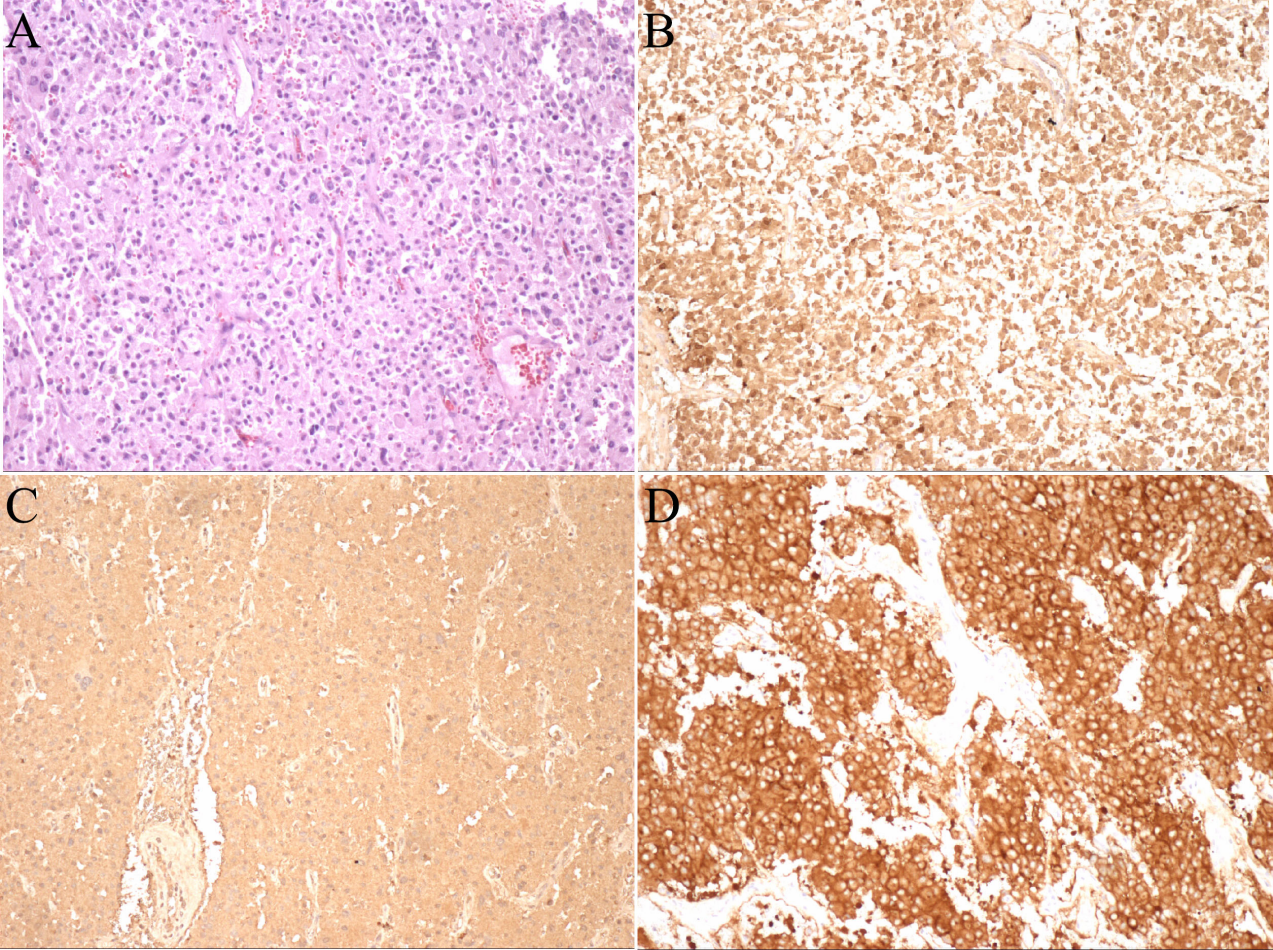
Pathological photos. **(A)** HE staining (×100); **(B)** Immunohistochemistry of soluble protein-100 (+); **(C)** Immunohistochemistry of chromogranin A (+); **(D)** Immunohistochemistry of synaptophysin (+).

The pathological diagnosis of the tumor was paraganglioma without adrenal involvement. Immunohistochemical analyses were as follows: soluble protein-100 (+), synaptophysin (+), chromogranin A (+), cytokeratin [CK (AE1/AE3)] (-), Ki-67 (index 2%), melanoma antigen (-), α-inhibin (-), succinate dehydrogenase B (+), O-6-methylguanine-DNA methyltransferase (-) (**Figure 3**). The patient was followed for 3 month after the operation, and there were no complications.

## Discussion

PGLs may involve or invade IVC (13). While some authors have reported successful resection of retrocaval PGLs (8–15), the location of the PGLs behind the retrohepatic segment of IVC made it much more difficult to expose and resect the tumor than those near renal vein level. A literature review of case reports of retrocaval PGLs was conducted using PubMed database. Only 11 cases have been reported (**Table 1**). Five of them had PGLs behind the retrohepatic segment of IVC (13, 14). Exposure of retrocaval PGLs should be careful, and mobilization of colon and duodenum is usually needed. Then sharp and blunt dissections of IVC and the right renal vein could help expose targeted tumors. However, if PGLs are located much higher and behind the retrohepatic segment of IVC, these may be not sufficient to expose the masses. Jia et al. reported a case of malignant PGL located behind the retrohepatic segment of the IVC in 2013 (14). Open surgery was chosen, and resection of the left lateral lobe of the liver was performed for exposure of the tumor. Soejima et al. also summarized 4 cases of PGLs/PHEOs behind retrohepatic IVC (13). All patients underwent open surgery. For the three cases with maximum diameters ranging from 5.5-7 cm, infrahepatic IVC (just proximal to the right renal vein) and suprahepatic IVC were initially controlled. While for the case with a much larger diameter of 16.7 cm, the infrahepatic IVC was temporarily transected to help expose the tumor, and was reconstructed after resection of the tumor. In addition to assistance of exposure, if there is extensive invasion of IVC, vena cava repair or reconstruction may also be required (10). If the IVC is completely occluded due to compression and invasion, the IVC may be ligated after tumor resection (10).

**Table 1:** Eleven previous cases of retrocaval PGLs.

No.	Reference	Sex	Age	Location	Size	Treatment	Incision	Assistance for exposure	Follow-up
**1**	Jia, 2013 (14)	Male	60	Behind the retrohepatic segment of IVC	8×6×4 cm	Open surgery (the mass, the left lateral lobe of the liver, a portion of the caudate lobe of the liver, and the gallbladder**)**	Right subcostal incision	Resection of the hepatic left lateral lobe	Discharged on postoperative day 21; died 6 months after surgery
**2**	Soejima, 2017 (13)	Female	43	Behind the retrohepatic IVC	6.0 cm	Open surgery	Bilateral subcostal incision with a midline extension	–	Discharged on postoperative day 11; alive 1370 days after surgery
**3**	Soejima, 2017 (13)	Male	20	Behind the retrohepatic IVC	5.5 cm	Open surgery	Bilateral subcostal incision with a midline extension	–	Discharged on postoperative day 12; alive 1308 days after surgery
**4**	Soejima, 2017 (13)	Male	45	Behind the retrohepatic IVC	16.7 cm	Open surgery	Bilateral subcostal incision with a midline extension	The infrahepatic IVC was temporally transected; the right lobe of the liver was mobilized to the left lobe with the transected IVC	Discharged on postoperative day 11; alive 867 days after surgery
**5**	Soejima, 2017 (13)	female	66	Behind the retrohepatic IVC	7.0 cm	Open surgery	Bilateral subcostal incision with a midline extension	–	Discharged on postoperative day 27; alive 314 days after surgery
**6**	Khawaja, 2013 (15)	Male	24	Retrocaval	4×4 cm	Open surgery	Midline incision	–	Discharged on postoperative day 6; free of haematuria and other complications on 4 months of follow-up
**7**	Nozaki, 2010 (8)	Male	58	Retrocaval	47 mm	Laparoscopic resection	A 12-mm trocar was placed near the level of the umbilicus, two other 12-mm trocars were placed in the ipsilateral abdomen	An additional 5-mm port was placed in the midline caudal to the xyphoid for liver retraction	Resumed oral intake and were ambulating within 2 days
**8**	Nozaki, 2010 (8)	Male	56	Retrocaval	44 mm	Laparoscopic resection	A 12-mm trocar was placed near the level of the umbilicus, two other 12-mm trocars were placed in the ipsilateral abdomen	An additional 5-mm port was placed in the midline caudal to the xyphoid for liver retraction	Resumed oral intake and were ambulating within 2 days
**9**	Bourke, 2002 (10)	Male	10	Retrocaval, extended from the level of the renal vein inferiorly to the bifurcation of the IVC	12×14 cm	Open surgery	–	The IVC was ligated at the level of the renal veins and at the caval bifurcation	Discharged 10 days post-primary resection; remained tumor-free at 4-year follow-up
**10**	Marshall, 2012 (11)	Male	28	Retrocaval, behind the right renal vein	35×30×30 mm	Open surgery	A midline laparotomy with right subcostal extension	–	Discharged on postoperative day 7
**11**	Alrasheedi, 2010 (9)	Male	24	Retrocaval, below the right renal vein	27 mm	Robotic-assisted surgery	A 12 mm port placed at 3 cm above Umbilicus, two 8 mm ports on anterior axillary line, and a 5 mm ports was placed for the first assistant	–	Normal at 24 months follow-up

Of the eleven cases reviewed, two underwent laparoscopic surgery and one underwent robotic-assisted surgery. Nozaki et al. found that the laparoscopic approach provided excellent exposure, allowing proper identification of the tumor’s origin and its relationship to surrounding structures (8). The robotic approach may be particularly appropriate for resection of paragangliomas in demanding localization (9). On the other hand, minimally invasive surgery may also help patients recover faster. The two cases in Nozaki’s report resumed oral intake and ambulation in two days after surgery, and the patient in our case report recovered within one day after surgery. In this case, we reported a difficult PGL also behind the retrohepatic segment of the IVC that was successfully resected by laparoscopy. There was no tumor invasion to the liver or IVC in this case, and laparoscopy provided a sufficient view without preconditioning the liver or IVC. Separation of the tumor and important vessels or tissues could be performed successfully. Evaluation of imaging and 3D printing may also help identify and separate tumor, surrounding tissures, and important vessels.

## Conclusion

We report a rare case of PGL that lies behind the retrohepatic segment of the IVC. The PGL was successfully resected by laparoscopy without IVC injury or partial liver resection. This case demonstrates that laparoscopic surgery may be helpful in tumor accessibility, and may be tried for resection of PGLs behind the retrohepatic segment of the IVC in appropriate cases.

## Ethics statement

Written informed consent was obtained from the patient for publication of this case report and any accompanying images.

## Author contributions

WW participated in the writing of the manuscript. JW contributed to the editing of the manuscript. JD, HL and ZJ served as scientific advisors. All authors contributed to the article and approved the submitted version.

## Supplementary material

Supplementary Figure 1 The right lateral peritoneum was opened to expose IVC and the tumor. The tumor was covered by IVC completely (1, right kidney; 2, IVC).

Supplementary Figure 2 The tumor area was further exposed by elevating the liver (1, tumor; 2, IVC; 3, portal vein).

Supplementary Figure 3 The important large vessels were protected by vessel loops. The loops could help for vessel traction and tumor exposure (1, tumor; 2, IVC; 3, portal vein; 4, left renal vein).

Supplementary Figure 4 The tumor was separated from IVC, and the regurgitant veins of the tumor were severed (1, tumor; 2, IVC; 3, portal vein).

Supplementary Figure 5 The adrenal gland was excised along with the tumor (arrow, central vein of adrenal gland; 1, IVC; 2, right adrenal gland).

Supplementary Figure 6 The arterial vessel from the aorta for tumor blood supply was isolated and cut off (arrow, the arterial vessel; 1, tumor).

Supplementary Figure 7 The tumor was separated from the surrounding tissue and resected completely (1, tumor; 2, IVC; 3, right adrenal gland).

Supplementary Figure 8 Specimen of the tumor (6×3.8×3.2cm of mass with 2.5×0.8×0.5cm of adrenal gland).
