# Supplementary figures and images for: Laparoscopic resection of a paraganglioma behind the retrohepatic segment of the inferior vena cava: a case report and literature review
Source: Front Endocrinol (Lausanne). 2023 Jul 17;14:1171045. doi: 10.3389/fendo.2023.1171045 (PMC10389038; doi:10.3389/fendo.2023.1171045)

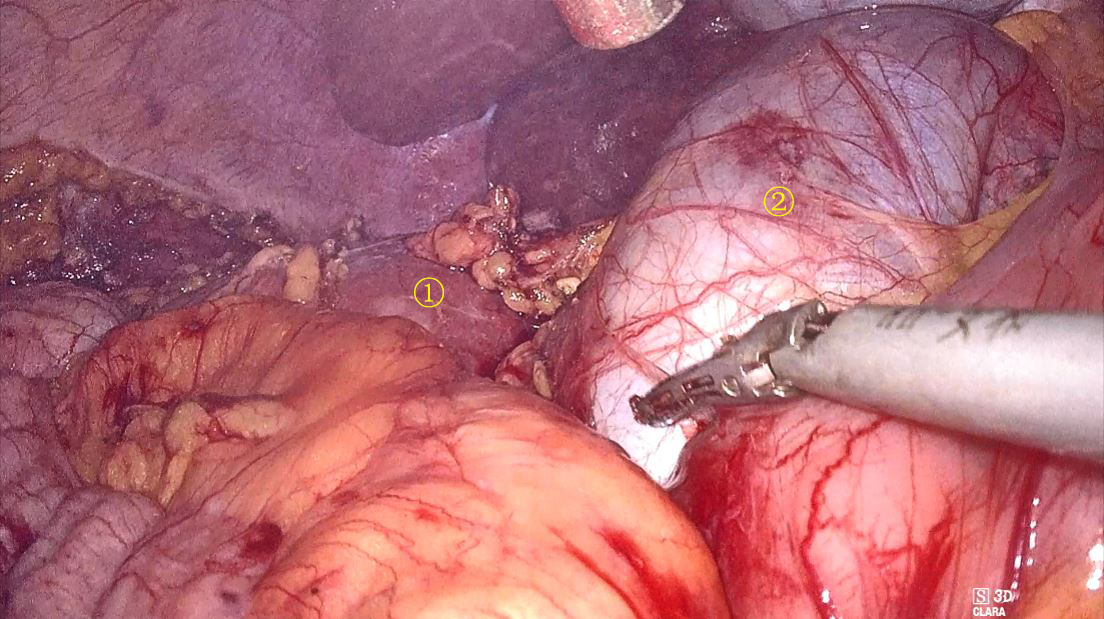

Supplement: Supplementary Figure 1 — The right lateral peritoneum was opened to expose IVC and the tumor. The tumor was covered by IVC completely (1, right kidney; 2, IVC). [file Image_1.tif]

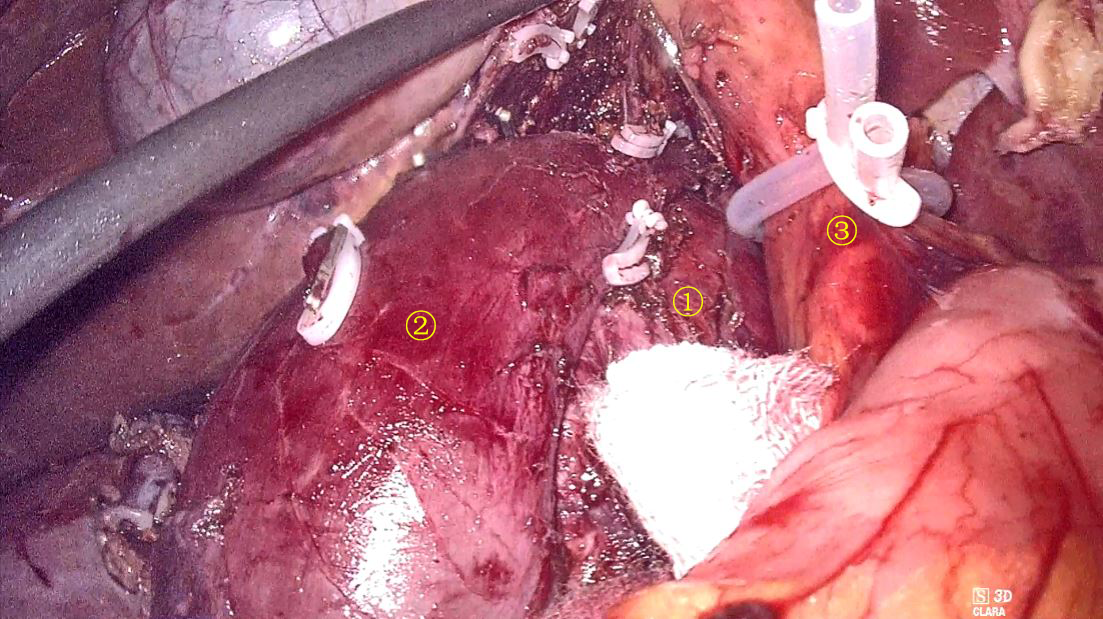

Supplement: Supplementary Figure 2 — The tumor area was further exposed by elevating the liver (1, tumor; 2, IVC; 3, portal vein). [file Image_2.tif]

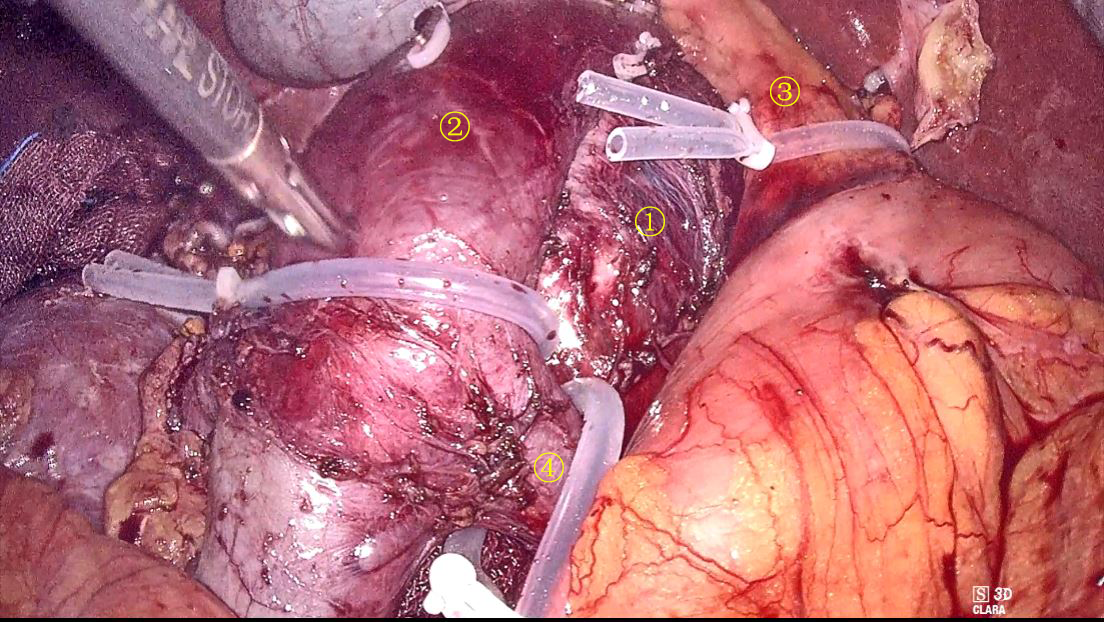

Supplement: Supplementary Figure 3 — The important large vessels were protected by vessel loops. The loops could help for vessel traction and tumor exposure (1, tumor; 2, IVC; 3, portal vein; 4, left renal vein). [file Image_3.tif]

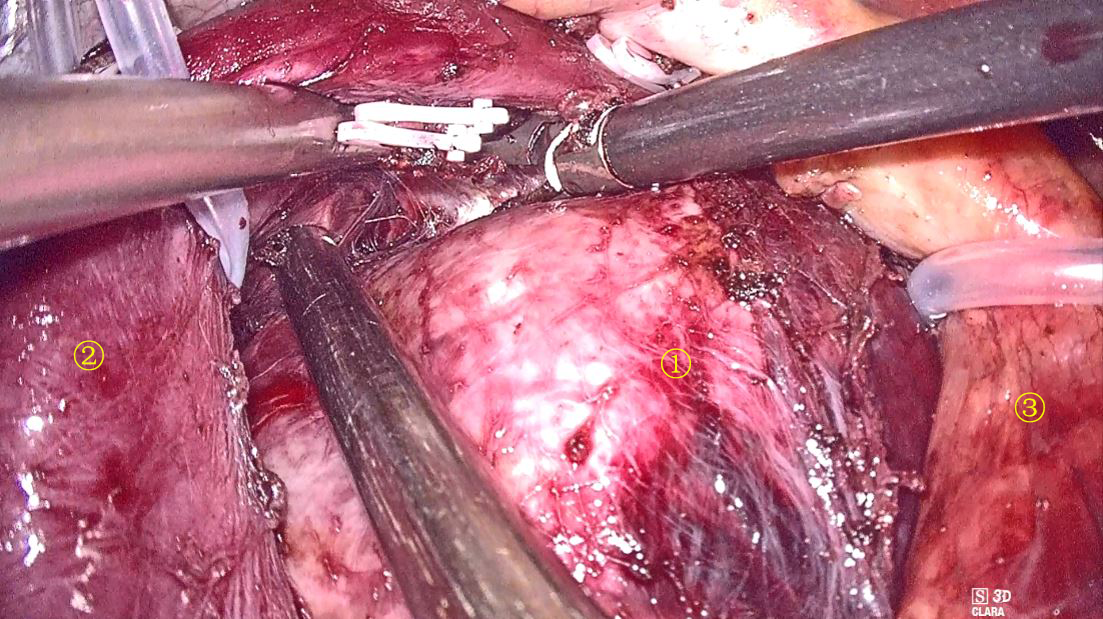

Supplement: Supplementary Figure 4 — The tumor was separated from IVC, and the regurgitant veins of the tumor were severed (1, tumor; 2, IVC; 3, portal vein). [file Image_4.tif]

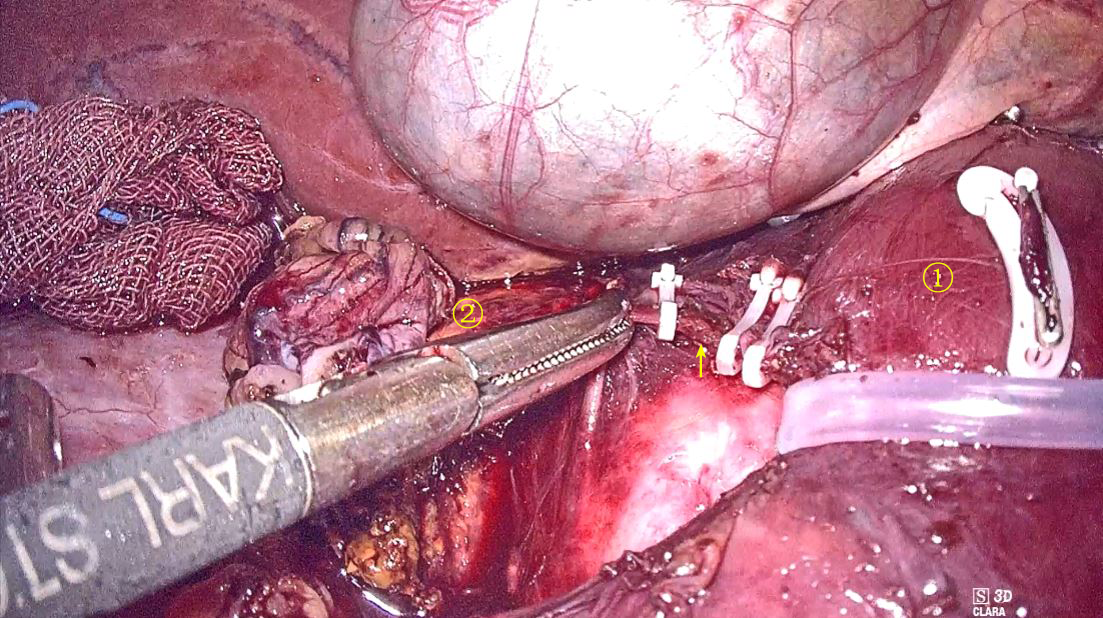

Supplement: Supplementary Figure 5 — The adrenal gland was excised along with the tumor (arrow, central vein of adrenal gland; 1, IVC; 2, right adrenal gland). [file Image_5.tif]

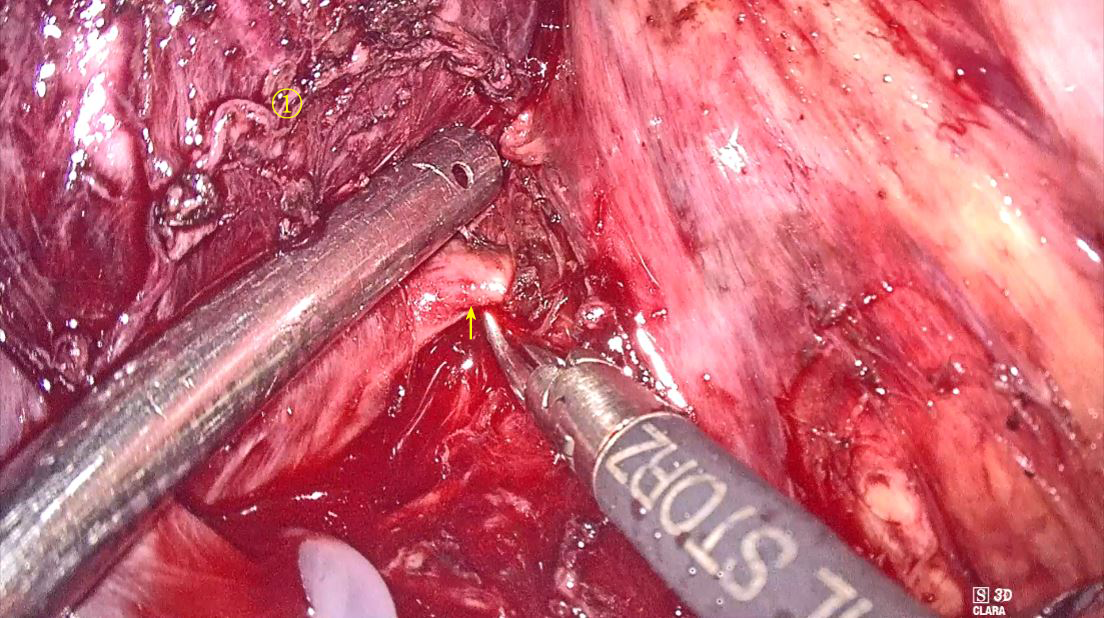

Supplement: Supplementary Figure 6 — The arterial vessel from the aorta for tumor blood supply was isolated and cut off (arrow, the arterial vessel; 1, tumor). [file Image_6.tif]

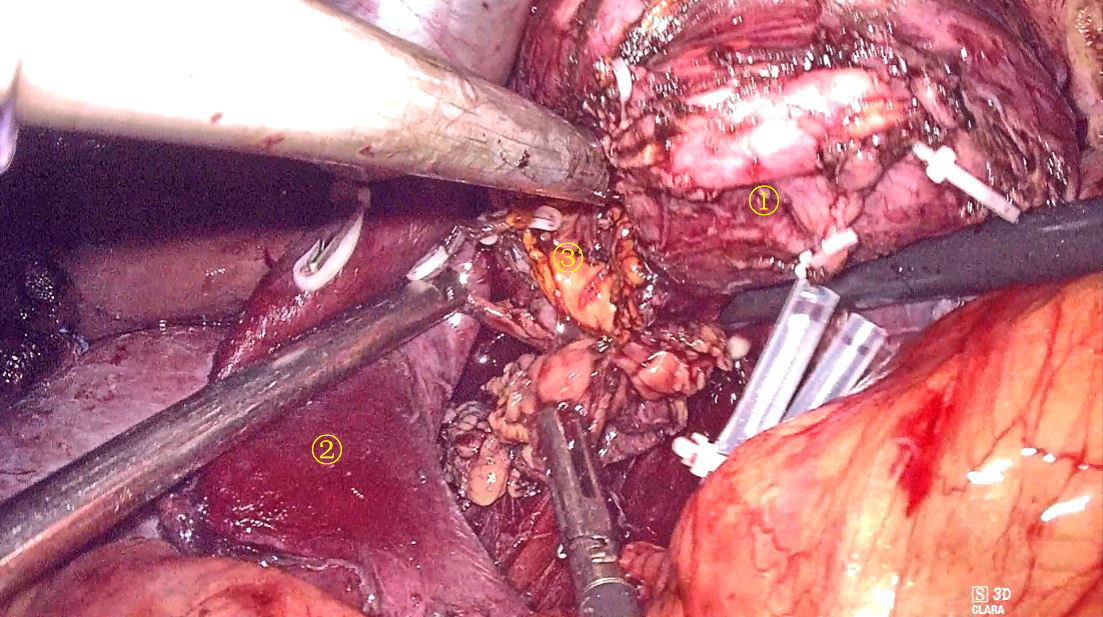

Supplement: Supplementary Figure 7 — The tumor was separated from the surrounding tissue and resected completely (1, tumor; 2, IVC; 3, right adrenal gland). [file Image_7.tif]

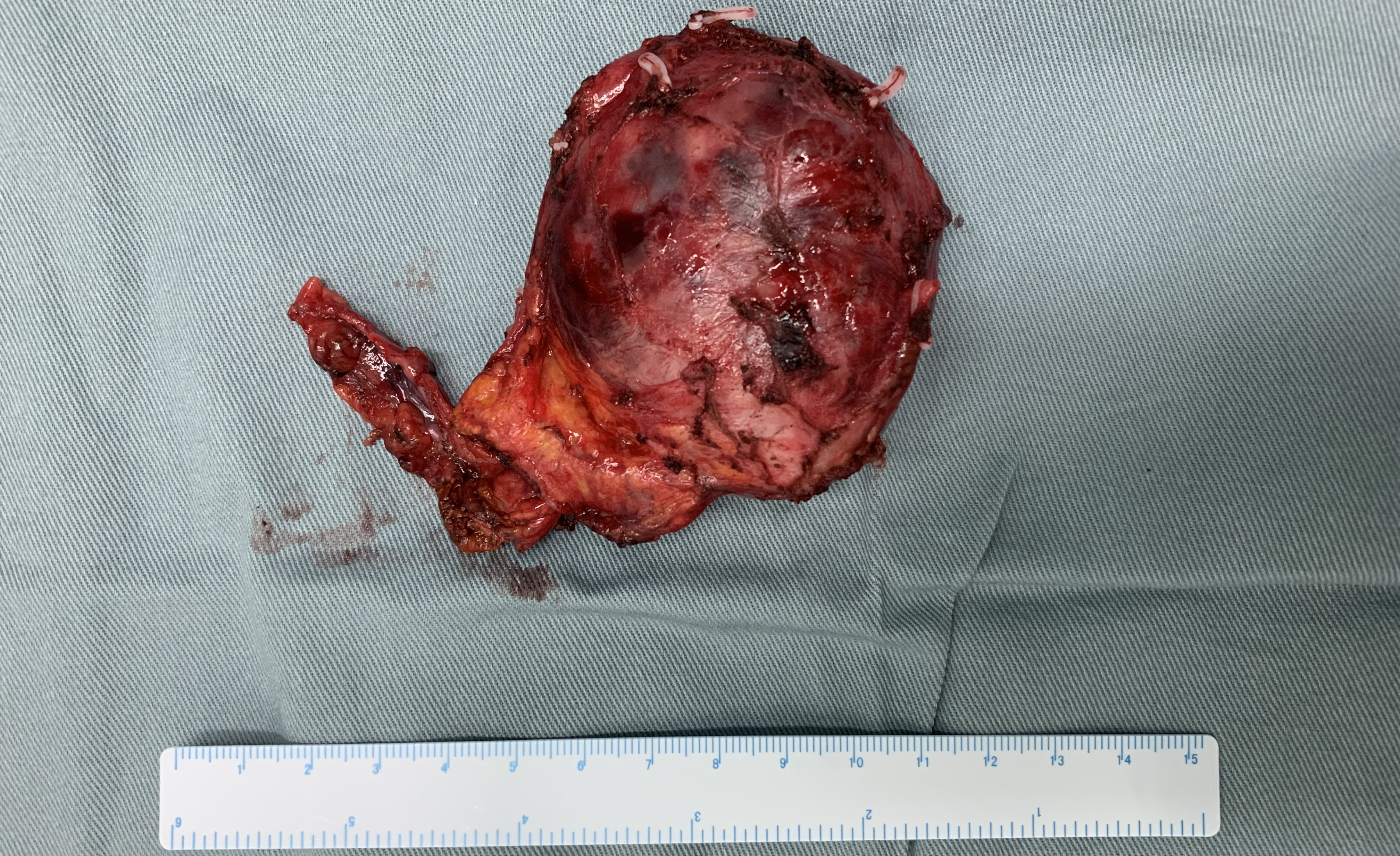

Supplement: Supplementary Figure 8 — Specimen of the tumor (6×3.8×3.2cm of mass with 2.5×0.8×0.5cm of adrenal gland). [file Image_8.tif]
